# Polypharmacy Is Associated With Slow Gait Speed and Recurrent Falls in Older People With HIV

**DOI:** 10.1093/cid/ciad782

**Published:** 2023-12-26

**Authors:** Priya Kosana, Kunling Wu, Katherine Tassiopoulos, Scott Letendre, Qing Ma, Robert Paul, Ronald Ellis, Kristine M Erlandson, Shelli F Farhadian

**Affiliations:** Department of Epidemiology of Microbial Diseases, Yale School of Public Health, New Haven, Connecticut, USA; Center for Biostatistics in AIDS Research, Harvard T. H. Chan School of Public Health, Boston, Massachusetts, USA; Department of Epidemiology, Harvard T. H. Chan School of Public Health, Boston, Massachusetts, USA; Department of Medicine, University of California San Diego, San Diego, California, USA; Department of Psychiatry, University of California San Diego, San Diego, California, USA; School of Pharmacy and Pharmaceutical Sciences, University of Buffalo, Buffalo, New York, USA; Department of Psychological Sciences, University of Missouri–St.Louis, St. Louis, Missouri, USA; Department of Psychiatry, University of California San Diego, San Diego, California, USA; Department of Neurosciences, University of California San Diego, San Diego, California, USA; Department of Medicine, University of Colorado Anschutz Medical Campus, Aurora, Colorado, USA; Department of Epidemiology of Microbial Diseases, Yale School of Public Health, New Haven, Connecticut, USA; Department of Medicine, Section of Infectious Diseases, Yale School of Medicine, New Haven, Connecticut, USA

**Keywords:** HIV, polypharmacy, hyperpolypharmacy, gait speed, falls

## Abstract

**Background:**

Older people with human immunodeficiency virus (HIV, PWH) are prone to using multiple medications due to higher rates of medical comorbidities and the use of antiretroviral therapy (ART). We assessed the prevalence and clinical impact of polypharmacy among PWH.

**Methods:**

We leveraged clinical data from the AIDS Clinical Trials Group A5322 study “Long-Term Follow-up of Older HIV-infected Adults: Addressing Issues of Aging, HIV Infection and Inflammation” (HAILO). We included PWH aged ≥40 years with plasma HIV RNA levels <200 copies/µL. We assessed the relationship between polypharmacy (defined as the use of 5 or more prescription medications, excluding ART) and hyperpolypharmacy (defined as the use of 10 or more prescription medications, excluding ART) with slow gait speed (less than 1 meter/second) and falls, including recurrent falls.

**Results:**

Excluding ART, 24% of study participants had polypharmacy and 4% had hyperpolypharmacy. Polypharmacy was more common in women (30%) than men (23%). Participants with polypharmacy had a higher risk of slow gait speed (odds ratio [OR] = 1.78; 95% confidence interval [CI] = 1.27–2.50) and increased risk of recurrent falls (OR = 2.12; 95% CI = 1.06–4.23). The risk for recurrent falls was further increased in those with hyperpolypharmacy compared with those without polypharmacy (OR = 3.46; 95% CI = 1.32–9.12).

**Conclusions:**

In this large, mixed-sex cohort of PWH aged ≥40 years, polypharmacy was associated with slow gait speed and recurrent falls, even after accounting for medical comorbidities, alcohol use, substance use, and other factors. These results highlight the need for increased focus on identifying and managing polypharmacy and hyperpolypharmacy in PWH.

## Teaser

Polypharmacy is prevalent among PWH. Slow gait speed and recurrent falls, both predictors of poor outcomes, are exacerbated in PWH with polypharmacy, even after accounting for other contributing factors.

The average age of people with human immunodeficiency virus (HIV, PWH) has increased with the widespread use of antiretroviral therapy (ART). Individuals aged 50–54 years now make up the largest age group of PWH [1]. This demographic shift has been accompanied by an increased burden of multimorbidity among PWH, with a 3-fold higher rate of multimorbidity in older (aged ≥50 years) vs younger PWH [2, 3]. Alongside the rise in multimorbidity comes increased polypharmacy, or the simultaneous use of multiple medications. Polypharmacy occurs at a rate that is 2 to 4 times higher in PWH than in the general population [4–6] and is further pronounced with aging. Compared with younger PWH, PWH aged >50 years have higher rates of polypharmacy [7, 8].

Polypharmacy is linked with several adverse outcomes in the general population, even after adjusting for comorbidities as a potential confounder. These include increased rates of hospitalization, falls, cognitive decline, and adverse drug events [9–15]. Among older community-dwelling adults, polypharmacy is associated with poor physical function, including slow gait speed, a measure of frailty that is strongly linked to adverse outcomes, including later mortality in people without HIV [16, 17]. However, less is known about the relationship between polypharmacy and adverse outcomes in PWH and whether there are sex differences in polypharmacy in PWH after accounting for potential differences in comorbidities [18–21]. Prior studies of polypharmacy in PWH found associations between polypharmacy and adverse outcomes, including serious falls [22] and impaired cognition [19]. However, a key factor that limits our understanding of the relationship between polypharmacy and adverse outcomes in PWH with virologic suppression is the paucity of studies that have focused on large cohorts of mixed-sex, aging PWH on ART, with a detailed inventory of medication use alongside detailed accounting of comorbidities and other important confounding factors.

Here, we leveraged data from the AIDS Clinical Trials Group A5322 study “Long-Term Follow-up of Older HIV-infected Adults: Addressing Issues of Aging, HIV Infection and Inflammation” (HAILO), a large, multicenter, longitudinal cohort of older men and women with virologically suppressed HIV, to better define the prevalence and deleterious effects of polypharmacy after accounting for confounders, including comorbidity burden. We focused on 2 outcomes of interest: slow gait speed and recurrent falls. In the general population, slow gait speed (slower than 1 meter/second walk time [23]) predicts adverse prognoses in older adults (eg, hospitalization, all-cause mortality), even among well-functioning older adults, and as such is an important “pre-disability” measure of frailty [24, 25]. Falls, particularly recurrent (2 or more) falls, predict hospitalization, disability, and cognitive decline [26–29].

## METHODS

### Study Participants and Study Design

HAILO enrolled older (aged ≥40 years) men and women with HIV who received their first ART treatment in an ACTG clinical trial. HAILO was a longitudinal cohort study that included biannual study visits where fall assessments, prescription medications, and clinical events were collected. Frailty evaluations (including gait speed) and laboratory tests were conducted once a year. We included all HAILO study participants who had gait speed and/or a history of falls obtained and a plasma HIV-1 RNA <200 copies/µL. This cross-sectional analysis used data from the HAILO study entry visit. Falls were first assessed in HAILO at the first follow-up visit, which occurred 6 months after study entry.

### Outcomes

Gait speed was assessed as the time required to complete a 4-meter walk and averaged over 2 trials as a part of the Fried frailty phenotype [30]. Slow gait speed was defined as a walk speed slower than 1 meter/second [24]. The number of falls in the past 6 months (no falls, single fall, and recurrent [≥2] falls) was self-reported biannually through a questionnaire administered by interview.

### Exposure

Prescription medications were recorded biannually through medical chart review or participant self-report. Polypharmacy was defined as the use of 5 or more prescription medications; hyperpolypharmacy was defined as the use of 10 or more prescription medications [31]. We assessed outcome variables, both including and excluding ART, in our definition of polypharmacy. We evaluated polypharmacy as a binary variable (≥5 vs <5 medications) and hyperpolypharmacy as a categorical variable (≥10 vs 5–9 vs <5 medications). Additionally, we assessed the use of certain prescription drug classes that may be particularly deleterious and pose increased risk for potential drug–drug interactions. We also determined if any reported medications were included in the Beers Criteria, a list of medications where the possible side effects outweigh the potential benefits in individuals aged >65 years [32].

### Covariates

Alcohol use was recorded using the Alcohol Use Disorders Identification Test questionnaire. Light alcohol use was defined as fewer than 7 drinks per week for men and fewer than 3 drinks per week for women. Moderate alcohol use was defined as 7 to 14 drinks per week for men and 3 to 7 drinks per week for women. Heavy alcohol use was defined as 15 or more drinks per week for men and 8 or more drinks per week for women. Binge drinking (defined as more than 5 drinks in a row for men and more than 4 drinks in a row for women) also constituted heavy alcohol use. Cigarette use and substance use (cannabis, cocaine, heroin, amphetamines, other nonprescribed drugs) were assessed with a self-reported questionnaire. Substance use was coded as a 3-level variable: never, prior use, and current use.

The following comorbidities were abstracted from medical charts: diabetes, chronic kidney disease, cardiovascular disease, and chronic liver disease. Cardiovascular disease included coronary artery disease (with or without revascularization surgery), myocardial infarction, stroke/transient ischemic attack, angina, peripheral arterial disease, cardiomyopathy/heart failure, arrhythmia, deep vein thrombosis, and pulmonary embolism. Peripheral neuropathy was assessed with a clinical exam or abstracted from medical charts. Comorbidity burden was defined as the total number of comorbidities. Because use of medications to treat hypertension and mental health conditions was part of the definition, these comorbidities were not included in the analysis. Detailed information regarding more specific mental health conditions was not collected.

Race, ethnicity, years of education, and medical insurance were self-reported. Sex at birth was included as a covariate; gender was not available.

### Statistical Analyses

We calculated the prevalence of polypharmacy and hyperpolypharmacy overall and compared their prevalence and that of specific drug classes across subgroups. The Wilcoxon test and *χ*^2^ test were used for statistical comparisons across subgroups for continuous and discrete demographic and clinical variables, respectively. Comparisons in prescription medication use across subgroups were made using the Kruskal–Wallis test or *χ*^2^ test.

Logistic regression models were fit to examine associations between polypharmacy and slow gait speed. One model was fit with the binary polypharmacy variable, and a separate model was fit with the categorial variable (<5, 5–9, and ≥10 medications) in order to evaluate the association of hyperpolypharmacy specifically. Multinomial logistic regression models were fit to evaluate the association of polypharmacy and hyperpolypharmacy with single and recurrent falls. As noted above, separate models were fit with the binary and categorical polypharmacy variables. Covariates included in models as potential confounders were chosen based on their clinical significance and because they were associated with the outcomes of interest in univariate analysis. All gait speed models were adjusted for sex, race, ethnicity, age, comorbidity burden, education, CD4 nadir, substance use, alcohol use, medical insurance, and body mass index (BMI). All models for falls were adjusted for sex, race, age, comorbidity burden, substance use, and medical insurance. We evaluated for the presence of modification by sex on the above associations by including interaction terms between polypharmacy and sex in each of the multivariable models.

## RESULTS

### Polypharmacy in HAILO

A total of 977 participants were included. The demographic and clinical characteristics of the study participants are summarized in Table 1. The median age of the sample was 51 years (standard deviation [SD], 8), and participants had a median of 14 years (SD, 4) of education. Forty-nine percent of participants were White non-Hispanic, and 43% had private medical insurance. A majority of participants (74%) did not actively use tobacco or illicit substances. Peripheral neuropathy was the most common comorbidity (40%), followed by diabetes (12%) and chronic kidney disease (10%). Twenty-four percent of participants were prescribed 5 or more non-ART medications (Table 1); 4% were prescribed 10 or more non-ART medications. When ART was included in the total count of prescription medications, 44% had polypharmacy and 8% had hyperpolypharmacy. Excluding ART medications, polypharmacy increased with age; 36% of participants aged ≥60 years were prescribed 5 or more non-ART medications compared with 22% aged <60 years. When comparing PWH with polypharmacy to those without polypharmacy, those with polypharmacy had lower educational levels, lower rates of alcohol use and smoking, and higher rates of public (rather than private or Medicare) health insurance coverage.

**Table 1.** Baseline Demographics and Clinical Characteristics of HAILO Participants

Characteristic	Total	Polypharmacy	Non-Polypharmacy	*P* Value
	(N = 977)	(n = 236)	(n = 741)	
Demographics				
Age, y				
Median (Q1, Q3)	51 (46, 56)	53 (49, 58)	50 (45, 55)	<.001
<50	423 (43%)	69 (29%)	354 (48%)	<.001
50–59	402 (41%)	113 (48%)	289 (39%)	
60–64	90 (9%)	27 (11%)	63 (9%)	
>65	62 (6%)	27 (11%)	35 (5%)	
Sex				
Female	184 (19%)	55 (23%)	129 (17%)	.044
Male	793 (81%)	181 (77%)	612 (83%)	
Race				
White	636 (65%)	147 (62%)	489 (66%)	.299*
Non-White	341 (35%)	89 (38%)	252 (34%)	
Ethnicity				
Hispanic or Latino	195 (20%)	30 (13%)	165 (22%)	.001*
Not Hispanic or Latino	780 (80%)	206 (87%)	574 (78%)	
Education				
Median (Q1, Q3)	14 (12, 16)	13 (12, 16)	14 (12, 16)	.019
Alcohol use				
None/Light	703 (75%)	181 (80%)	522 (74%)	.05
Moderate/Heavy	232 (25%)	45 (20%)	187 (26%)	
Smoking				
Never/Prior	710 (74%)	163 (70%)	547 (75%)	.104
Current	253 (26%)	71 (30%)	182 (25%)	
Substance use (excluding smoking)				
Never/Prior	734 (79%)	180 (79%)	554 (79%)	.847
Current	197 (21%)	47 (21%)	150 (21%)	
Medical insurance				
None/Unknown	188 (19%)	31 (13%)	157 (21%)	<.001
Public	258 (26%)	84 (36%)	174 (23%)	
Private	416 (43%)	71 (30%)	345 (47%)	
Medicare	115 (12%)	50 (21%)	65 (9%)	
Body mass index				
Median (Q1, Q3)	27 (24, 31)	28 (24, 31)	27 (24, 31)	.173
Median (Q1, Q3)				
Human immunodeficiency virus characteristics				
CD4 nadir	197 (66, 304)	191 (46, 297)	198 (72, 307)	.189
CD4 cell count	630 (457, 828)	664 (451, 884)	616 (464, 823)	.125
Years on antiretroviral therapy	8 (4, 12)	11 (7, 13)	8 (4, 12)	<.001
Comorbidity				
Diabetes	118 (12%)	69 (29%)	49 (7%)	<.001
Kidney disease	102 (10%)	47 (20%)	55 (7%)	<.001
Liver disease	11 (1%)	6 (3%)	5 (1%)	.018
Cardiovascular disease	57 (6%)	34 (14%)	23 (3%)	<.001
Peripheral neuropathy	388 (40%)	119 (50%)	269 (36%)	<.001
History of any comorbidity	499 (51%)	173 (73%)	326 (44%)	<.001
Number of comorbidities	1 (0, 1)	1 (0, 2)	0 (0, 1)	<.001

^*^Chi-square test. All other comparisons made by Wilcoxon test.

### Sex Differences in Polypharmacy

Although women had a similar number of comorbidities, women were more likely to experience polypharmacy and hyperpolypharmacy than men (Table 2). Women aged 50–54 and 55–59 years were prescribed, on average, 3 non-ART prescription medications compared with only 2 among men in the same age ranges (Supplementary Figure 1). When examining specific drug classes, women reported higher prescription opioid use (16%) compared with men (8%; Table 2). Black women had the highest proportion of opioid use (21%), but use was higher among women compared with men across all race and ethnicity categories (Supplementary Table 1). Additionally, women were prescribed hormones at a greater rate than men. There was no difference in benzodiazepine, gabapentin, or pregabalin use in women vs men.

**Table 2.** Polypharmacy Differences in HAILO Participants by Sex

Characteristic	Total	Male	Female	*P* Value
	(N = 977)	(n = 793)	(n = 184)	
Number of non-ART prescription medications				
Median (Q1, Q3)	2 (1, 4)	2 (1, 4)	3 (1, 5)	.006
0	168 (17%)	143 (18%)	25 (14%)	.154
1–4	573 (59%)	469 (59%)	104 (57%)	
5–9	194 (20%)	150 (19%)	44 (24%)	
≥10	42 (4%)	31 (4%)	11 (6%)	
Number of prescription medications (non-ART + ART)				
Median (Q1, Q3)	4 (3, 6)	4 (3, 6)	5 (3, 7)	<001
1–4	541 (55%)	458 (58%)	83 (45%)	.008
5–9	354 (36%)	273 (34%)	81 (44%)	
≥10	82 (8%)	62 (8%)	20 (11%)	
Medication class				
Opioids	93 (10%)	63 (8%)	30 (16%)	<.001
Benzodiazepines	94 (10%)	78 (10%)	16 (9%)	.636
Hormones (estrogens, androgens, progesterone)	75 (8%)	54 (7%)	21 (11%)	.035
Gabapentin and pregabalin	81 (8%)	62 (8%)	19 (10%)	.266
Anticholinergics	37 (3.8%)	30 (3.8%)	7 (3.8%)	.989
Comorbidity				
Diabetes	118 (12%)	90 (11%)	28 (15%)	.147
Chronic kidney disease	102 (10%)	82 (10%)	20 (11%)	.833
Cardiovascular disease	57 (6%)	50 (6%)	7 (4%)	.192
Liver disease	11 (1%)	8 (1%)	3 (2%)	.472
Peripheral neuropathy	388 (40%)	317 (40%)	71 (39%)	.729
History of any recorded comorbidity	499 (51%)	409 (52%)	90 (49%)	.515
Waist circumference, cm				
Median (Q1, Q3)	96 (88, 104)	96 (88, 103)	97 (88, 109)	.033

Abbreviation: ART, antiretroviral therapy.

### Potential Inappropriate Medications in PWH Aged ≥ 65 Years

According to Beers Criteria, most (81%) participants aged >65 years were on at least 1 inappropriate medication (Table 3) [32]. For individuals who were prescribed at least 1 medication considered inappropriate by Beers Criteria, the median number of “inappropriate” medications was 2, with no significant differences observed by sex. Ninety-four percent of Black participants reported inappropriate medication use compared with 76% of White non-Hispanic and Hispanic participants (*P* = .16).

**Table 3.** Inappropriate Medication Use Among Study Participants Aged ≥ 65 Years

Characteristic	Total Participants Aged ≥65 Years (N = 62)	Participants With ≥ 1 Inappropriate Medication (n = 50)	*P* Value
Number of inappropriate medications			
Median (Q1, Q3)		2 (1, 3)	
Min, Max		1, 9	
Sex			
Male	51	41 (80%)	.914
Female	11	9 (82%)	
Race/ethnicity			
White Non-Hispanic	31	23 (74%)	.274
Black Non-Hispanic	16	15 (94%)	
Hispanic	15	12 (80%)	

### Polypharmacy and Slow Gait Speed in Older PWH

Forty percent of participants exhibited slow gait speed, defined as a 4-meter walk time greater than 4 seconds. This prevalence was higher among Hispanic individuals, those with public insurance, females, and participants aged ≥55 years. Additionally, individuals with slow gait speed had a lower median CD4+ T-cell count (614 cells/mm) compared with those without slow gait speed (642 cells/mm).

**Figure 1.**
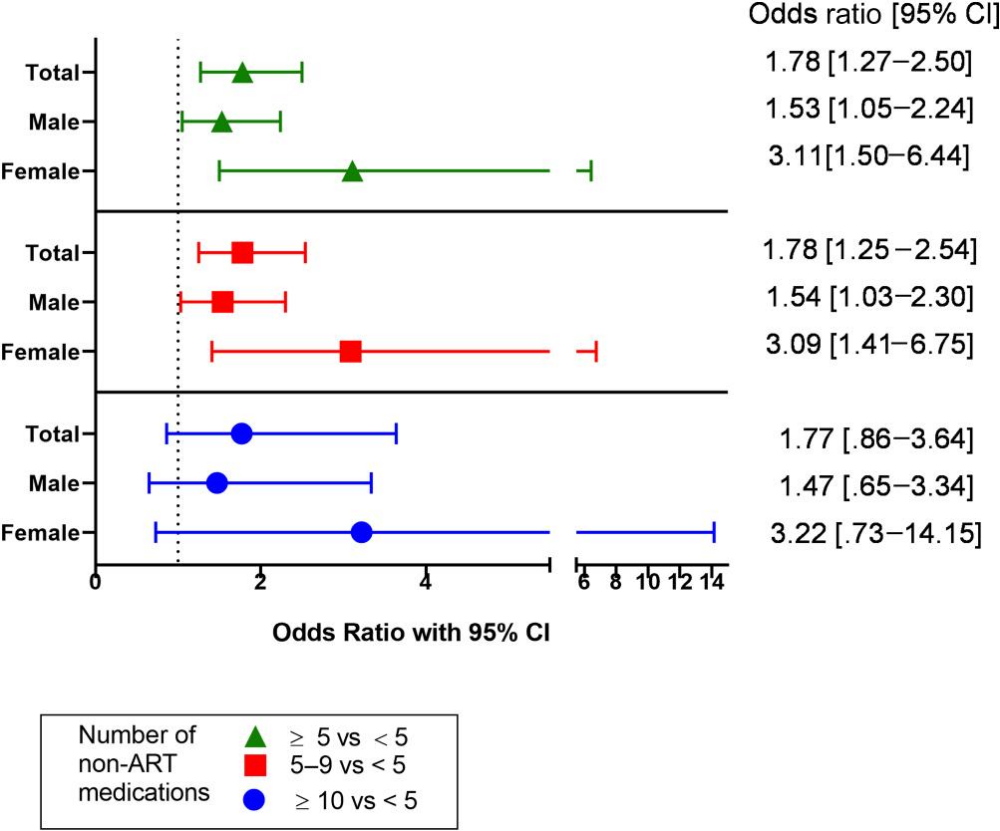
Odds ratio for slow gait speed in association with polypharmacy and hyperpolypharmacy in older people with human immunodeficiency virus (PWH). Comparisons are made for PWH with ≥5 vs <5 prescription medications (green), for 5–9 vs <5 prescription medications (red), and for ≥10 vs <5 medications (blue). ART was not included in the tally of prescription medications for this analysis. The gait speed model was adjusted for sex, race, ethnicity, age, comorbidity burden, education, CD4 nadir, substance use, alcohol use, medical insurance, and body mass index. Abbreviations: ART, antiretroviral therapy; CI, confidence interval.

Univariable models revealed that polypharmacy was associated with higher odds of slow gait speed (odds ratio [OR] = 2.16; 95% confidence interval [CI] = 1.61–2.91). Polypharmacy remained associated with slow gait speed in multivariable models that accounted for age, sex, race, ethnicity, total number of comorbidities, education, CD4 nadir, substance use, alcohol use, medical insurance, and BMI (OR = 1.78; 95% CI = 1.27–2.50; Figure 1). Hyperpolypharmacy did not further increase the risk for slow gait speed (OR = 1.77; 95% CI = .86–3.64). However, when ART medications were included in the analysis, hyperpolypharmacy conferred higher odds of slow gait speed than polypharmacy-negative individuals (OR = 1.77; 95% CI = 1.02–3.07; Supplementary Figure 2). There was no evidence of effect modification by sex on the association between polypharmacy and slow gait speed.

### Polypharmacy and Falls

**Figure 2.**
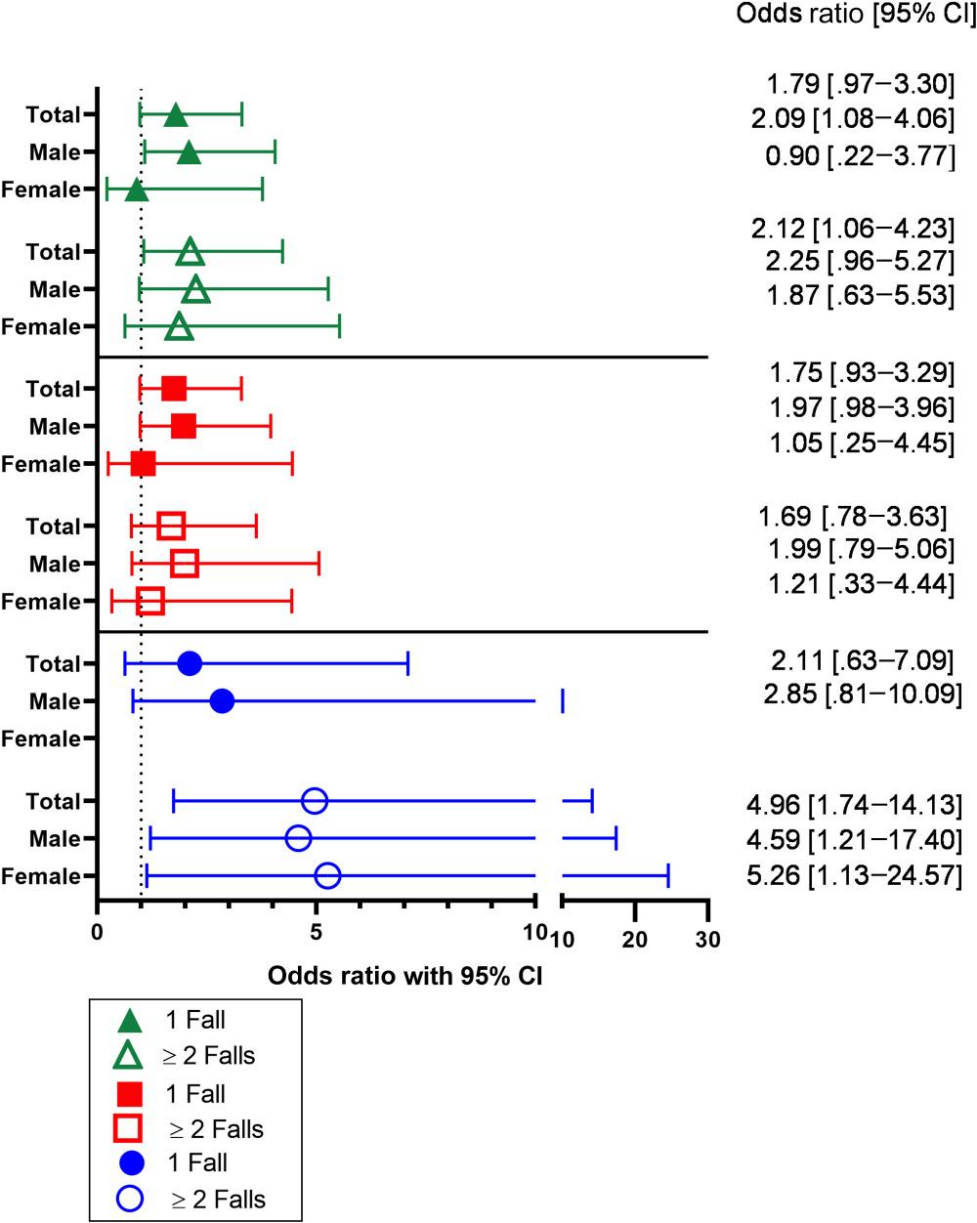
Odds ratio for single and recurrent falls in association with polypharmacy and hyperpolypharmacy in older people with human immunodeficiency virus (PWH). Closed icons indicate the odds of having 1 fall in the last 6 months; open icons indicate the odds of having 2 or more falls. Comparisons are made for PWH with ≥5 vs <5 prescription medications (green), for 5–9 vs <5 prescription medications (red), and for ≥10 vs <5 medications (blue). Antiretroviral therapy was not included in the tally of prescription medications for this analysis. The falls model was adjusted for sex, race, age, comorbidity burden, substance use, and medical insurance. Abbreviation: CI, confidence interval.

Twelve percent of HAILO participants reported at least 1 fall in the last 6 months; 7% reported 1 fall, and 5% reported recurrent (2 or more) falls. A higher prevalence of falls was associated with female sex, Hispanic ethnicity, public medical insurance, and participants aged ≥55 years. Among those who did experience a fall, 3.85% reported a fracture and 22% sought medical attention. Univariate analysis of polypharmacy was associated with 2.39 higher odds (95% CI = 1.56–3.67) of 1 or more falls compared with individuals without polypharmacy. In univariate models, polypharmacy was associated with 1.99 higher odds (95% CI = 1.15–3.46) of a single fall and 3.07 higher odds (95% CI = 1.65–5.73) of recurrent falls. In multivariable models, the association of polypharmacy was somewhat attenuated but remained associated with higher odds of experiencing recurrent falls (OR = 2.12; 95% CI = 1.06–4.23), and hyperpolypharmacy exacerbated this risk of recurrent falls (OR = 4.96; 95% CI = 1.74–14.13; Figure 2). These findings were consistent when ART was included in the number of medications defining polypharmacy and hyperpolypharmacy (Supplementary Figure 3). We found no evidence of effect modification by sex on the association between polypharmacy and single or recurrent falls.

## DISCUSSION

In this study, we examined polypharmacy and 2 adverse functional outcomes, slow gait speed and recurrent falls, in a large cohort of older men and women with suppressed HIV. We found a high polypharmacy rate, even when ART was removed from the count of medications. We also found that certain high-risk medications, including opioids, were more commonly used among women and that 81% of PWH aged ≥65 years were taking a medication with high potential for adverse effects. Most important, we found that polypharmacy, especially hyperpolypharmacy, was associated with poor outcomes, including higher odds of slow gait speed and recurrent falls, even after adjusting for multimorbidity and other confounders. Although previous studies showed that women had higher rates of both polypharmacy and comorbidities [33–37], ours is the first to report that this association remained statistically significant after adjusting for the number of comorbidities. This suggests that the adverse effects of polypharmacy are not simply due to an accumulation of medical conditions but rather may reflect potential toxicities and other effects of numerous simultaneous medications, in addition to the effects of comorbidities.

Prior studies reported an increased risk for falls among PWH compared with the general population, with PWH experiencing falls at a younger age compared with adults without HIV [38]. Our results indicate that polypharmacy and hyperpolypharmacy, in particular, are strongly associated with a higher risk for recurrent falls among PWH. This is especially worrisome since recurrent falls are more likely to be associated with serious injuries and increased morbidity and mortality in community-dwelling adults [28, 29, 39–41]. Moreover, our finding that polypharmacy was associated with slow gait speed may further impact the risk for falls in older PWH. In previous studies conducted in individuals without HIV, a slow gait speed was positively associated with multiple falls [17, 42]. Thus, efforts to pinpoint and reduce risk factors for polypharmacy among PWH, including the presence of specific comorbidities and social determinants of health, can potentially mitigate the risk of recurrent falls and associated functional decline.

Consistent with prior reports in the general population, we found that the prevalence of polypharmacy was higher in women than in men [35–37] (30% vs 23%), despite the fact that women had a similar comorbidity burden and had similar HIV-related factors (CD4+ T-cell count and CD4/CD8 ratio) as men in this cohort. Women in this study did differ from men by sociodemographic factors, including race (more likely to identify as Black) and medical insurance status (more likely to report using public insurance). This is consistent with prior studies of the general population where socioeconomic inequalities of polypharmacy are apparent. Socioeconomic status and education are inversely related to polypharmacy, largely due to the increased risk of multimorbidity and poor care coordination among populations of low socioeconomic status [43, 44]. Importantly, we were not able to account for mental health diagnoses in HAILO and for potential sex differences in mental health diagnoses and associated drug use. An inclusion of mental health disorders in our recorded comorbidities may have changed comorbidity burden between men and women since women with HIV experience a higher burden of mental health disorders than their male counterparts [45]. Further studies are needed to understand why women with HIV may be prescribed more non-ART medications compared with men and whether a higher prevalence of mental health conditions among women with HIV is an explanatory factor.

An important limitation of our study is that we were not able to account for all medical comorbidities, including hypertension and mental health disorders, as discussed above, since the diagnosis of certain conditions in HAILO relied on prescription drug usage. We also did not have access to information on medication adherence, dosage, or indication, which limits our understanding of the use of certain medications, including opioids. Although we adjusted for the total number of comorbidities, we did not assess the impact of specific comorbidities or the severity of specific comorbidities on polypharmacy and hyperpolypharmacy.

In summary, by using data collected from a large, multicenter, mixed-age, and mixed-sex cohort of people with HIV, we found that polypharmacy is prevalent among PWH. We found that polypharmacy is linked with both slow gait speed and recurrent falls, even after accounting for multiple medical conditions that are the basis for prescription medication use and that may themselves lead to an increased risk of slow gait speed or falls, such as peripheral neuropathy and alcohol use disorder. By using data from this large cohort, we were able to stratify results by sex and found that, despite having an equal number of comorbidities, women were prescribed more medications than men, including more opioids. Attempts to reduce frailty and associated adverse outcomes in PWH will therefore need to address polypharmacy, particularly the appropriateness of opioid prescriptions in women with HIV.

These findings have several important implications. First, they suggest that there may be a need for increased attention to medication management among PWH, particularly among women and older individuals. This could include efforts to reduce polypharmacy and inappropriate medication use and increased monitoring for potential drug–drug interactions. Additionally, the finding that polypharmacy was associated with higher odds of slow gait speed suggests that polypharmacy may have negative impacts on physical function among PWH.

In the future, it may be useful to further explore the factors that contribute to polypharmacy and inappropriate medication use among PWH, as well as the potential impacts of these issues on health outcomes. Additionally, interventions aimed at reducing polypharmacy and inappropriate medication use among PWH could be developed and tested.

## Supplementary Data

Supplementary materials are available at *Clinical Infectious Diseases* online. Consisting of data provided by the authors to benefit the reader, the posted materials are not copyedited and are the sole responsibility of the authors, so questions or comments should be addressed to the corresponding author.

## Supplementary Material

ciad782_Supplementary_Data

## Notes

***Financial support.*** This work was supported by grants from the NIH, National Institute of Allergy and Infectious Diseases (under awards UM1 AI068634, UM1 AI068636, UM1 AI069494, UM1 AI106701, K23MH118999, and P30AG021342-20S1) and the National Institute on Aging (K24AG082527 to K. M. E. and R01AG063659 (to Q. M. and S. L.). Q. M. reports support for this work from Gilead Science, Merck, and ViiV.
