# Supplementary material for: Polypharmacy Is Associated With Slow Gait Speed and Recurrent Falls in Older People With HIV
Source: Clin Infect Dis. 2023 Dec 26;78(6):1608–16. doi: 10.1093/cid/ciad782 (PMC11175684; doi:10.1093/cid/ciad782)
Supplement: ciad782_Supplementary_Data [file ciad782_supplementary_data.pptx]

## Slide 1
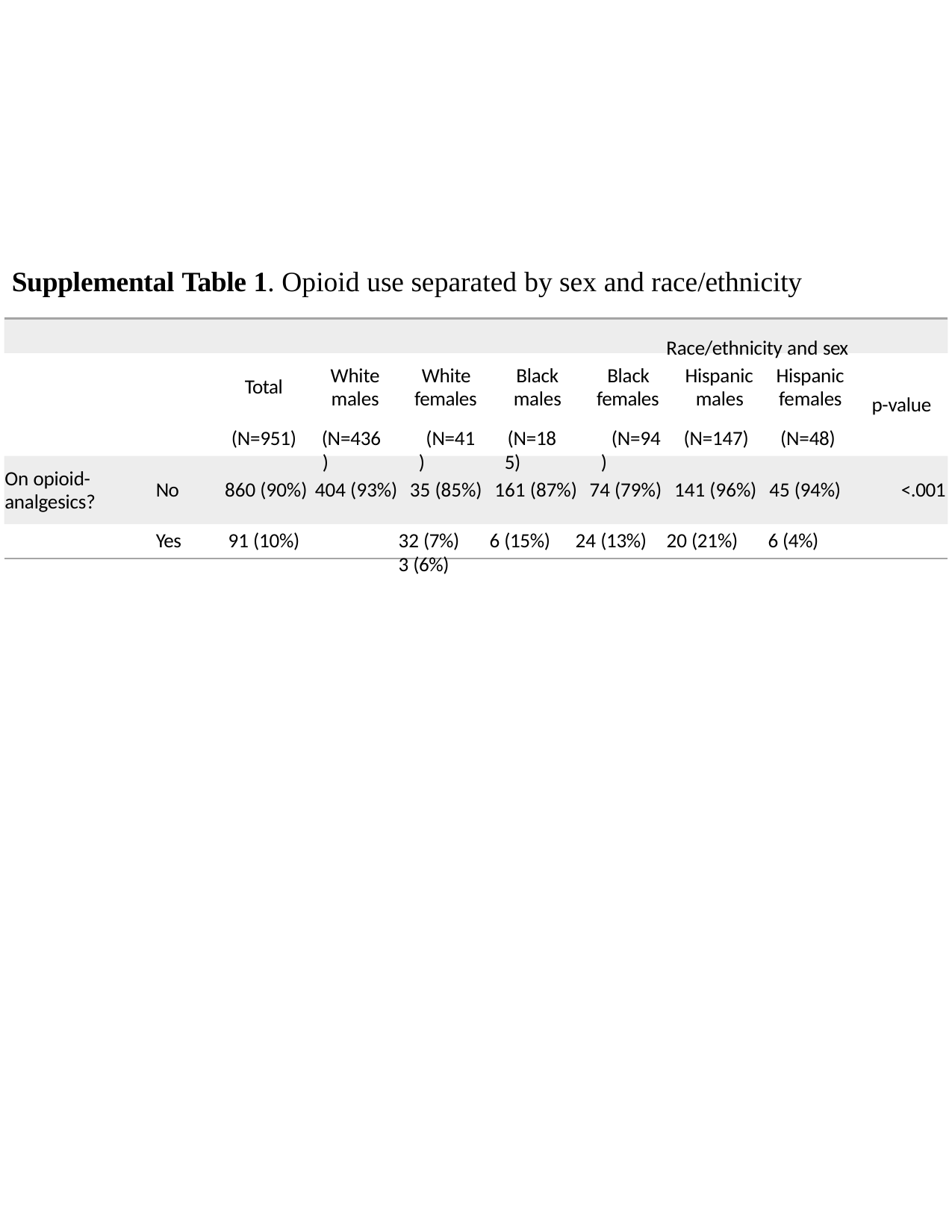

Supplemental Table 1. Opioid use separated by sex and race/ethnicity
Race/ethnicity and sex
White males
(N=436)
White females
(N=41)
Black males
(N=185)
Black females
(N=94)
Hispanic males
(N=147)
Hispanic females
(N=48)
Total
p-value
(N=951)
On opioid- analgesics?
No	860 (90%) 404 (93%) 35 (85%) 161 (87%) 74 (79%) 141 (96%) 45 (94%)
91 (10%)	32 (7%)	6 (15%)	24 (13%)	20 (21%)	6 (4%)	3 (6%)
<.001
Yes

## Slide 2
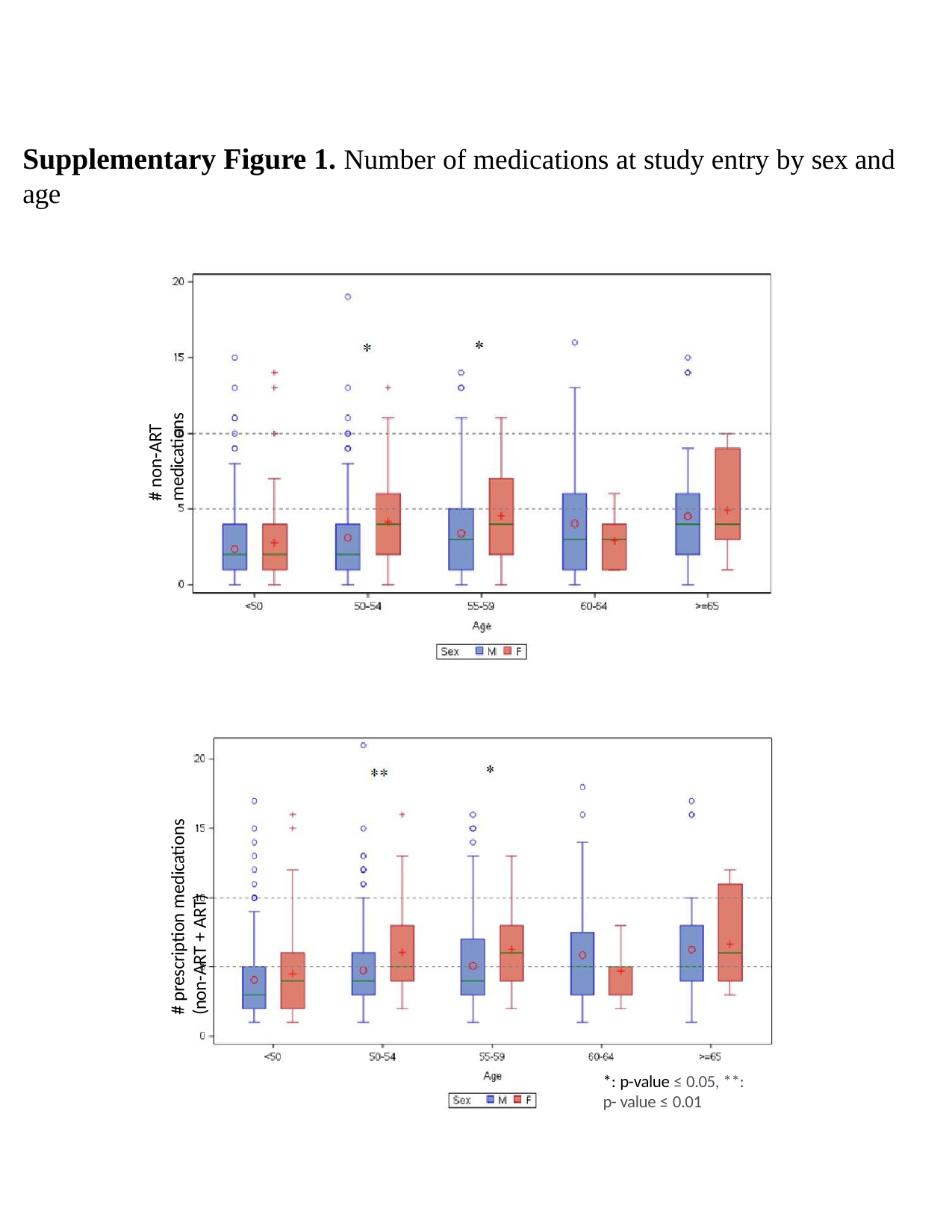

Supplementary Figure 1. Number of medications at study entry by sex and age
# non-ART medications
# prescription medications (non-ART + ART)
*: p-value ≤ 0.05, **: p- value ≤ 0.01

## Slide 3
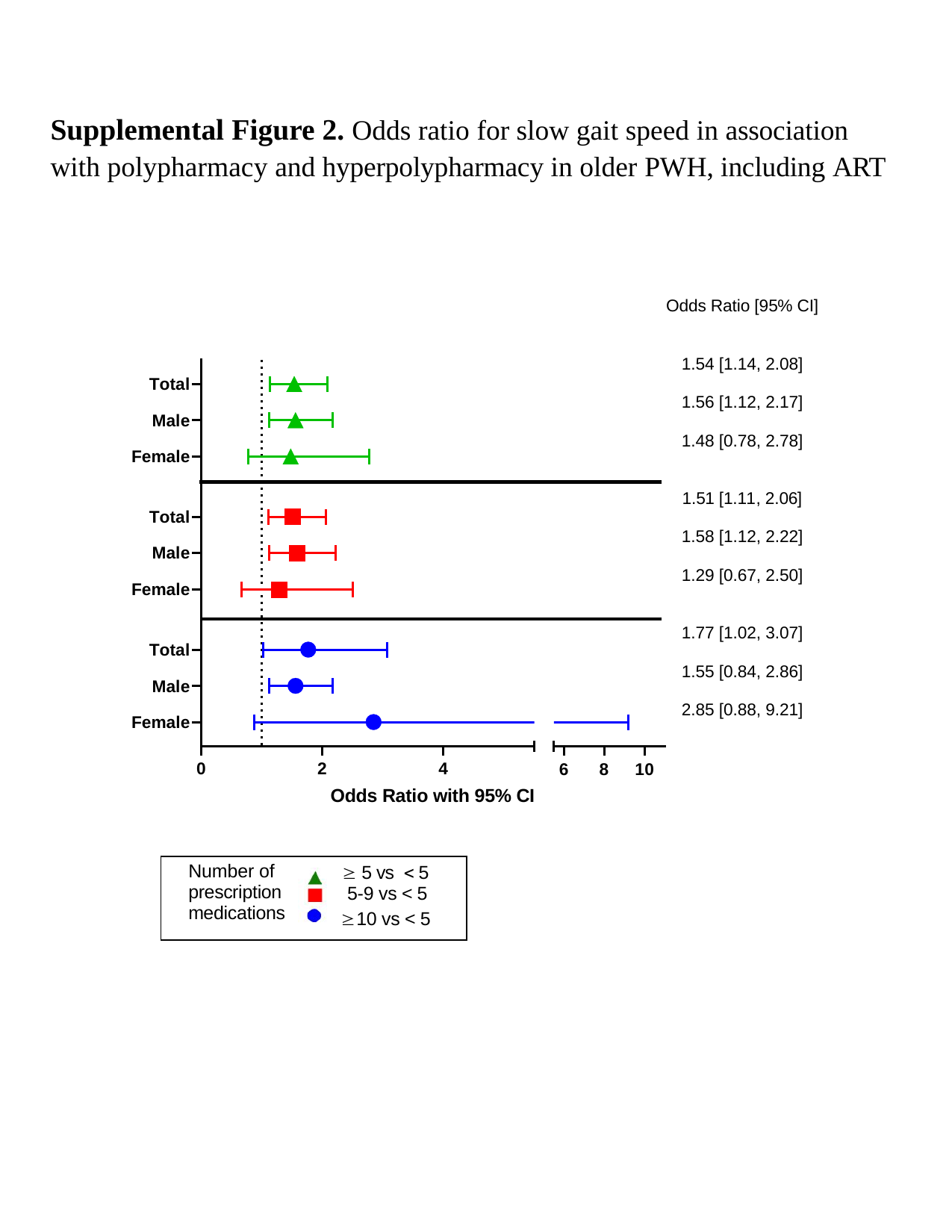

Supplemental Figure 2. Odds ratio for slow gait speed in association with polypharmacy and hyperpolypharmacy in older PWH, including ART

## Slide 4
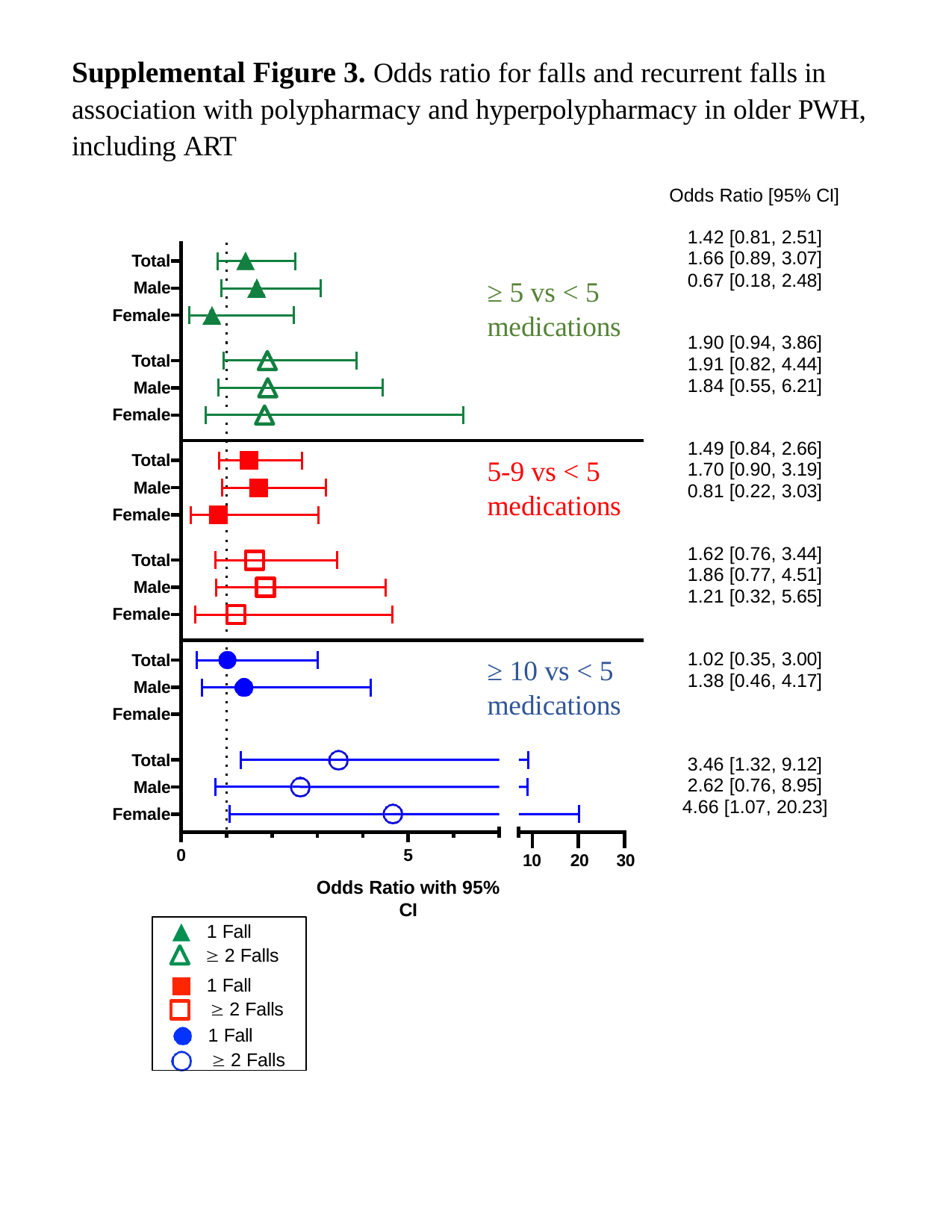

Supplemental Figure 3. Odds ratio for falls and recurrent falls in association with polypharmacy and hyperpolypharmacy in older PWH, including ART
Odds Ratio [95% CI]
1.42 [0.81, 2.51]
1.66 [0.89, 3.07]
0.67 [0.18, 2.48]
Total Male Female
≥ 5 vs < 5
medications
1.90 [0.94, 3.86]
1.91 [0.82, 4.44]
1.84 [0.55, 6.21]
Total Male Female
1.49 [0.84, 2.66]
1.70 [0.90, 3.19]
0.81 [0.22, 3.03]
Total Male Female
5-9 vs < 5
medications
1.62 [0.76, 3.44]
1.86 [0.77, 4.51]
1.21 [0.32, 5.65]
Total Male Female
Total Male Female
1.02 [0.35, 3.00]
1.38 [0.46, 4.17]
≥ 10 vs < 5
medications
Total Male Female
3.46 [1.32, 9.12]
2.62 [0.76, 8.95]
4.66 [1.07, 20.23]
5
Odds Ratio with 95% CI
0
10	20	30
1 Fall
≥ 2 Falls
1 Fall
≥ 2 Falls
1 Fall
≥ 2 Falls
